# A Matlab toolbox for scaled-generic modeling of shoulder and elbow

**DOI:** 10.1038/s41598-021-99856-y

**Published:** 2021-10-21

**Authors:** Ehsan Sarshari, Yasmine Boulanaache, Alexandre Terrier, Alain Farron, Philippe Mullhaupt, Dominique Pioletti

**Affiliations:** 1 Automatic Control Laboratory, Ecole Polytechnique Fédérale de Lausanne (EPFL), Lausanne, Switzerland; 2 Laboratory of Biomechanical Orthopedics, Ecole Polytechnique Fédérale de Lausanne (EPFL), Lausanne, Switzerland; 3 Department of Orthopedics and Traumatology, University Hospital Centre and University of Lausanne (CHUV), Lausanne, Switzerland

**Keywords:** Biomedical engineering, Mechanical engineering

## Abstract

There still remains a barrier ahead of widespread clinical applications of upper extremity musculoskeletal models. This study is a step toward lifting this barrier for a shoulder musculoskeletal model by enhancing its realism and facilitating its applications. To this end, two main improvements are considered. First, the elbow and the muscle groups spanning the elbow are included in the model. Second, scaling routines are developed that scale model’s bone segment inertial properties, skeletal morphologies, and muscles architectures according to a specific subject. The model is also presented as a Matlab toolbox with a graphical user interface to exempt its users from further programming. We evaluated effects of anthropometric parameters, including subject’s gender, height, weight, glenoid inclination, and degenerations of rotator cuff muscles on the glenohumeral joint reaction force (JRF) predictions. An arm abduction motion in the scapula plane is simulated while each of the parameters is independently varied. The results indeed illustrate the effect of anthropometric parameters and provide JRF predictions with less than 13% difference compared to in vivo studies. The developed Matlab toolbox could be populated with pre/post operative patients of total shoulder arthroplasty to answer clinical questions regarding treatments of glenohumeral joint osteoarthritis.

## Introduction

### Motivation

There exist several musculoskeletal models for the human upper extremity, e.g.^1–10^. They provide useful predictions of the joint and muscle forces that cannot be measured non-invasively^11^.

In a musculoskeletal model of the upper extremity any major joints or muscles should not be neglected^11,12^. Otherwise, its clinical applications will be limited. Some of the above-mentioned upper extremity models^3,10^ only include an outstretched arm. Therefore, the elbow and the muscle groups spanning the elbow and the shoulder (biceps and triceps) were neglected. Several studies highlighted the importance of the elbow on the upper extremity kinematics and dynamics, e.g.^13,14^. Biceps and triceps were also shown to play a crucial role in the stability of the glenohumeral joint^15,16^.

On the other hand, a vast majority of these models were developed based on anthropometric data of a single subject. Therefore, they are called generic models. Given anthropometric variabilities among subjects, the models face limitations in predicting inter-individual differences^17^. It is not yet straightforward to personalize all the anthropometric data used in a model to any subject. Obtaining these data is both time-consuming and expensive^17,18^. Alternatively, scaling techniques are used that scale a generic model to a specific subject (scaled-generic modeling)^13,19^.

### State-of-the-art

Given the complex multiple degrees of freedom (DOF) kinematics of the elbow, there is no consensus in the literature concerning its modeling^14,20^. Its multiple DOF result in two distinguished movements, including extension/flexion and pronation/supination. A very simplistic cardanic joint was used to model the two movements of the elbow^21^. But, it was not realistic, given that contrary to cardanic joints, the rotation axes of the elbow are not perpendicular^22^. Other studies used two non-perpendicular hinge joints^13,23–26^. In^23–25^, a single body was yet considered to represent two bone segments of the forearm (ulna and radius) neglecting the interplay between ulna and radius^27^. The sophisticated interplay of the ulna and the radius during forearm pronation/supination was studied using MRI imaging^28,29^. More complicated elbow models were developed considering a closed-kinematic chain^26,27,30^. They provided detailed representations of the elbow kinematics. But, they required more individualized parameters that could not be readily obtained.

Several aspects of an upper extremity model must be scaled, in order to adapt it to a specific subject. This includes bone segments inertial properties (BSIP), skeletal morphologies, and muscles architectures^31^.

#### Scaling BSIP

Predictive equations for BSIP were developed by investigating the BSIP of large groups of living subjects and cadavers^32–34^. They were commonly used to scale BSIP in upper extremity models^5,13,35^. However, they require further adjustments to accurately scale BSIP in the three dimensional (3D) space. The predictive equations of^36,37^ were adjusted by^38^ to provide 3D applicable predictive equations to scale BSIP.

#### Scaling skeletal morphologies

This includes scaling the positions of all bony landmarks based on which a generic model is developed. Isotropic scaling factors equally scaled the skeletal morphologies of a generic model in 3D^5,13,39^. However, this implied an unrealistic uniform scaling between different individuals^32^ and could also lead to discontinuous kinematics^39^. Anisotropic scaling factors provided more realistic scaling^40,41^. But, they could cause non-anatomical configurations of the scapula relative to ribcage^42^. This was improved by relaxing a kinematic constraint used to force the scapula medial boarder to glide over the ribcage^40^. However, the scapula motions could be compromised. A more advanced scaling based on an optimal scaling of the ribcage was introduced in^35^.

Several studies reported the effects of glenoid inclination/version on model force predictions and joint translations^43–46^. However, the available models do not allow adapting them to an understudy subject.

#### Scaling muscles architectures

This includes scaling muscle origins/insertions, wrapping objects, and musculotendon parameters such as physiological cross section areas (PCSA)^47^.

Muscle origins/insertion: Three different approaches were used to scale muscles origins/insertions, namely linear bone mapping^48,49^, anisotropic bone morphing^50–52^, and statistical bone morphing^53,54^. A linear bone mapping was defined using the positions of few bony landmarks on a specific subject^48,49^. The resulting mapping was used to approximate the muscle origins/insertions by rotating their corresponding origins/insertions from a generic model. This was considerably improved by considering not only rotations but also displacements and bone deformations in anisotropic bone morphing methods^50–52^. However, they required complete bone morphologies of each subject that could be obtained only by expensive imaging techniques. Statistical bone morphing methods were developed by investigating statistical shape variations of bones^53,54^. They also required at least part of the bone morphologies from each subject.

Muscle wrapping objects: The paths taken by muscles are not always a straight line from their origins to their insertions. They instead wrap around their underlying bones and soft tissues that are approximated by wrapping objects. Scaling the wrapping objects was not addressed at least explicitly in the literature. However, they can considerably alter muscle moment arms and model force predictions^55,56^.

Musculotendon parameters: PCSA was scaled by comparing the total muscle bulk of a subject to that of a generic model^57,58^. However, an MRI scan was required that might affect the practical use of this method. The body mass index (BMI)^59^ was also used to scale PCSA^13^, although considering a linear relationship between BMI and PCSA was controversial^60^. A predictive equation based on the BMI was introduced in^61^ that defined muscle percentage of the body composition. Scaling of other musculotendon parameters such as tendon length were addressed by using isotropic scaling factors^62^.

### Research method

Therefore, the aim of this study is to develop a scaled-generic musculoskeletal model of the shoulder and the elbow. The model is used to evaluate the effects of subject’s gender, height, weight, glenoid inclination, and rotator cuff (RC) PCSAs on the JRF. To this end, the elbow is included into our previously developed shoulder model^10^. Scaling routines are developed to scale model’s BSIP, skeletal morphologies, and muscles architectures according to a subject’s gender, height, weight. It also allows adapting the model’s glenoid inclination/version. An abduction motion in the scapula plane is simulated. The results are presented in terms of the JRF predictions along the arm abduction angle. Also, the model is developed as a Matlab toolbox with a graphical user interface (GUI) to facilitate its clinical applications.

## Methods

A kinematic model of the elbow is developed (“Elbow kinematics” section). Build on the resulting kinematic model, a dynamic model of the elbow is derived (“Elbow dynamics” section). The dynamic model of the elbow together with fourteen muscles spanning the elbow are integrated into the shoulder model (“Integration of the elbow model into the shoulder model” section). Our approaches in scaling the developed shoulder and elbow model are detailed (“Model scaling” section). The shoulder and elbow Matlab toolbox is also introduced (“Shoulder and elbow Matlab toolbox” section). Finally, a parameter study is performed to evaluate the model (“Parameter study” section). All figures reported here were specifically created with Matlab 2015b (www.mathworks.com), completed with images of MRI and bone surfaces using Amira V6 (www.thermofisher.com), and combined with Illustrator CC 2015 (www.adobe.com).

### Elbow kinematics

The kinematic development of our shoulder model including the thorax, the clavicle, the scapula, and an outstretched arm has been described in^10,63^. It was developed from MRI scans of the right shoulder of a healthy male subject (29 years, 186 cm, and 85.5 kg). It had seven DOF attributing to three ball-and-socket joints and two holonomic constraints. The joints were associated to the sternoclavicular, the acromioclavicular, and the glenohumeral (GH) joints. The constraints were considered for restricting the scapula to always glide on the ribcage. Here we focus on extending the kinematic model to incorporate the elbow.

**Figure 1:**
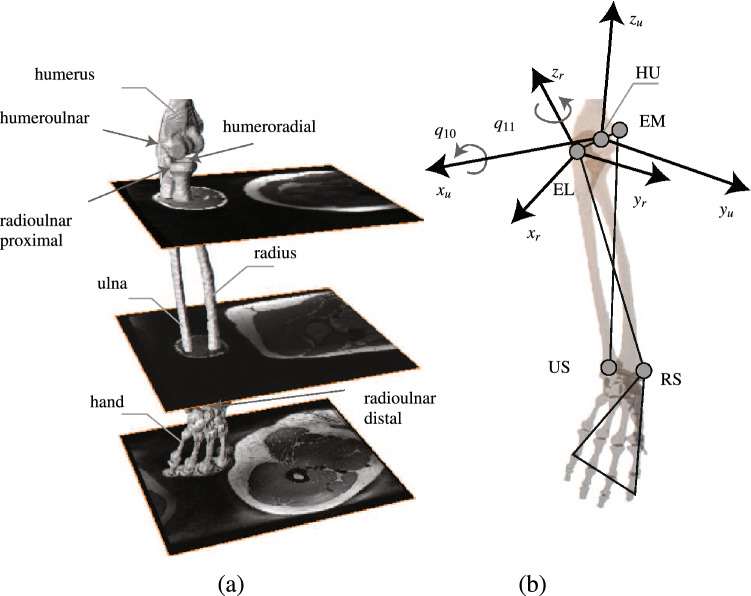
Modeling the elbow kinematics. (**a**) MRI scans of the forearm from the same subject are used to define the surface boundaries of the ulna and the radius. The hand is assumed to be rigidly tied to the radius. The elbow consists of 4 anatomical joints including humeroulnar, radioulnar proximal/distal, and humeroradial joints. (**b**) Two non-perpendicular hinge joints are considered to replicate the elbow motion. To construct the bone-fixed frames, 3 landmarks namely EM, HU, and EL are borrowed from the humerus. Two landmarks namely US and RS are also used from ulna and radius, respectively. $\{{{\varvec{HU}}},{\hat{x}}_{u},{\hat{y}}_{u},{\hat{z}}_{u}\}$ and $\{{{\varvec{EL}}},{\hat{x}}_{r},{\hat{y}}_{r},{\hat{z}}_{r}\}$ are the ulna and the radius frames, respectively. The joint coordinates are considered to be coincide with the bone-fixed frames. Two generalized coordinate $q_{10}$ and $q_{11}$ are used to uniquely define elbow flexion/extension and pronation/supination, respectively.

Surface boundaries of the forearm bones are obtained from MRI scans of the same subject (Fig. 1a). The ulna and the radius are considered as two segments. The hand is assumed to be rigidly tied to the radius by neglecting the carpal joint. Two DOFs are considered attributing to two non-perpendicular hinge joints. The two hinge joints replicate the kinematics of four anatomical joints, including the humeroulnar, the radioulnar proximal/distal, and the humeroradial joints. They allow simulating forearm flexion/extension and pronation/supination. Three non-collinear bony landmarks are required to uniquely define the spatial configuration of each bone segment in the 3D space^64^. Only one landmark can be discerned on each one of the ulna and the radius, i.e. the ulnar styloid process (US) and the radial styloid process (RS), respectively (Fig. 1b). Therefore, three bony landmarks on the humerus are borrowed, including the lateral epicondyle (EL) and the medial epicondyle (EM) and their middle point (HU). Two bone-fixed frames are defined for the ulna and the radius using these landmarks as follows:

- Ulna bone-fixed frame: $\{{{\varvec{O}}}_{u},{\hat{x}}_{u},{\hat{y}}_{u},{\hat{z}}_{u}\}$
  1 $$\begin{aligned} \begin{array}{ll} {{\varvec{O}}}_{u} \equiv {}_{t}{{\varvec{HU}}}\\ {\hat{x}}_{u} = {}_{t}{{\varvec{EL}}}-{}_{t}{{\varvec{HU}}} \implies {\hat{x}}_{u} = {{\hat{x}}_{u}}/{|{\hat{x}}_{u}|}\\ {\hat{y}}_{u} = ({}_{t}{{\varvec{US}}} - {}_{t}{{\varvec{EL}}})\times ({}_{t}{{\varvec{EM}}} - {}_{t}{{\varvec{EL}}}), \implies {\hat{y}}_{u} = {{\hat{y}}_{u}}/{|{\hat{y}}_{u}|}\\ {\hat{z}}_{u} = {\hat{x}}_{u}\times {\hat{y}}_{u}\\ {}^{t}_{u}R = [{\hat{x}}_{u}~{\hat{y}}_{u}~{\hat{z}}_{u}] \end{array} \end{aligned}$$
- Radius bone-fixed frame: $\{{{\varvec{O}}}_{r},{\hat{x}}_{r},{\hat{y}}_{r},{\hat{z}}_{r}\}$
  2 $$\begin{aligned} \begin{array}{ll} {{\varvec{O}}}_{r} \equiv {}_{t}{{\varvec{EL}}}\\ {\hat{z}}_{r} = {}_{t}{{\varvec{EL}}}-{}_{t}{{\varvec{US}}} \implies {\hat{z}}_{r} = {{\hat{z}}_{r}}/{|{\hat{z}}_{r}|}\\ {\hat{y}}_{r} = ({}_{t}{{\varvec{EL}}} - {}_{t}{{\varvec{RS}}})\times ({}_{t}{{\varvec{RS}}} - {}_{t}{{\varvec{US}}}), \implies {\hat{y}}_{r} = {{\hat{y}}_{r}}/{|{\hat{y}}_{r}|}\\ {\hat{x}}_{r} = {\hat{y}}_{r}\times {\hat{z}}_{r}\\ {}^{t}_{r}R = [{\hat{x}}_{r}~{\hat{y}}_{r}~{\hat{z}}_{r}] \end{array} \end{aligned}$$

where the symbol $\times$ denotes the cross product of vectors, and ${{\varvec{O}}}_{u}$ and ${{\varvec{O}}}_{r}$ are the origins of the ulna and the radius frames, respectively. The left hand side subscript *t* specifies that the landmarks are expressed in the thorax (inertial) frame, and ${}^{t}_{u}R$ and ${}^{t}_{r}R$ are also the rotation matrices from the ulna and the radius frames to the thorax frame, respectively.

The joint coordinates are considered to be aligned with the bone-fixed frames (Fig. 1b). Two generalized coordinates ($q_{10}$ and $q_{11}$) are used to uniquely define each joint configuration. More specifically, $q_{10}$ and $q_{11}$ represent the elbow flexion/extension and pronation/supination, respectively. The rotation matrices in terms of the generalized coordinates can be obtained as Eq. (3).

3 $$\begin{aligned} \begin{array}{ll} {}^{t}_{u}R = {}^{t}_hR~ {}^h_{u}R_f~ R_{{\hat{x}}_{u}}(q_{10})\\ {}^{t}_{r}R = {}^{t}_{u}R~ {}^u_{r}R_f~ R_{{\hat{z}}_{r}}(q_{11}) \end{array} \end{aligned}$$

where ${}^{t}_hR$ is the rotation matrix from the humerus frame to the thorax frame. The rotation matrices ${}^h_{u}R_f$ and ${}^u_{r}R_f$ are used to align the ulna and the radius frames to their proximal bone frames. Once they are defined for any configuration of the ulna and the radius, they remain unchanged. They are obtained as ${}^h_{u}R_f = {}^{t}_h{R_f}^{T}{}^{t}_{u}{R_f}$ and ${}^u_{r}R_f = {}^{t}_{u}{R_f}^{T}{}^{t}_{r}{R_f}$, where the right-hand side rotation matrices are defined using Eqs. (1) and (2) for an arbitrary configuration of the shoulder and the elbow. The rotation matrices for flexion/extension and pronation/supination ($R_{{\hat{x}}_{u}}$ and $R_{{\hat{z}}_{r}}$) are defined in Eq. (4).

4 $$\begin{aligned} \begin{array}{ll} R_{{\hat{x}}_{u}}= \begin{bmatrix} 1 &{}\quad 0 &{}\quad 0 \\ 0 &{}\quad \cos {q_{10}}&{}\quad -\sin {q_{10}}\\ 0 &{}\quad \sin {q_{10}}&{}\quad \cos {q_{10}} \end{bmatrix}\\ R_{{\hat{z}}_{r}}= \begin{bmatrix} \cos {q_{11}} &{}\quad -\sin {q_{11}} &{}\quad 0 \\ \sin {q_{11}} &{}\quad \cos {q_{11}}&{}\quad 0\\ 0 &{}\quad 0&{}\quad 1 \end{bmatrix} \end{array} \end{aligned}$$

It is worth mentioning that, introduction of ${}^h_{u}R_f$ and ${}^u_{r}R_f$ deviates from the ISB recommendations^65^. Because, the ISB requires inherent alignment of the ulna and the radius frames relative to their proximal bones. The inherent alignment however results in non-physiological configurations of the forearm relative to humerus^66^.

The following forward kinematics map ($\xi _{\mathrm{elbow}}$) is obtained using Eqs. (1) to (4). It defines ${}_{t}{{\varvec{US}}}$ and ${}_{t}{{\varvec{RS}}}$ for given $q_{10}$, $q_{11}$, ${}_{t}{{\varvec{EL}}}$, and ${}_{t}{{\varvec{EM}}}$ (Eq. 5). It is incorporated in the previously developed forward kinematic map of the shoulder model to provide a complete representation of the upper extremity kinematics.

5 $$\begin{aligned} \begin{array}{ll} \xi _{\mathrm{elbow}} : C_{s} \subset R^{2} \mapsto W_{s} \subset R^3\\ \xi _{\mathrm{elbow}} (q_{10}(t),~ q_{11}(t)) = {{\varvec{x}}}_j(t),~~ j=\{{\text {US}},\;{\text {RS}}\} \end{array} \end{aligned}$$

where $C_{s}$ and $W_{s}$ denote the elbow’s coordinate and work spaces^64^, and ${{\varvec{x}}}_{\text {US}}(t)\equiv {}_{t}{{\varvec{US}}}$, and ${{\varvec{x}}}_{\mathrm{RS}}(t)\equiv {}_{t}{{\varvec{RS}}}$.

### Elbow dynamics

Mass and inertial properties are attributed to the ulna and the radius according to^1^. The mass and inertia of the hand are also added to radius using parallel axis theorem^64^. The centers of mass (${{\varvec{CG}}}$) of the ulna and the radius are defined as

6 $$\begin{aligned} \begin{array}{ll} {{\varvec{CG}}}_{u} = \frac{1}{2}~ {}^{t}_{u}R~ {}_{u}{{\varvec{US}}} + {}_{t}{{\varvec{HU}}}\\ {{\varvec{CG}}}_{r} = \frac{1}{2}~ {}^{t}_{r}R~ {}_{r}{{\varvec{RS}}} + {}_{t}{{\varvec{EL}}} \end{array} \end{aligned}$$

where the left-hand side subscripts *u* and *r* specify that the landmarks are expressed in the ulna and the radius frames, respectively.

The angular velocities of the ulna (${{\varvec{\omega }}}_{u}$) and the radius (${{\varvec{\omega }}}_{r}$) in their bone-fixed frames are given by

7 $$\begin{aligned} \begin{array}{ll} {{\varvec{\omega }}}_{u} = R_{{\hat{x}}_{u}}^{T} ~ {}^h_{u}R_f^{T} ~ {{\varvec{\omega }}}_h + [{\dot{q}}_{10}~0~0]^{T}\\ {{\varvec{\omega }}}_{r} = R_{{\hat{z}}_{r}}^{T} ~ {}^u_{r}R_f^{T} ~ {{\varvec{\omega }}}_{u} ~+ [0~0~{\dot{q}}_{11}]^{T} \end{array} \end{aligned}$$

where ${{\varvec{\omega }}}_h$ is the humerus angular velocity.

The Lagrangians of the ulna (${\mathscr {L}}_{u}$) and the radius (${\mathscr {L}}_{r}$) are defined in terms of their kinetic and potential energies (Eq. 8).

8 $$\begin{aligned} \begin{array}{ll} {\mathscr {L}}_{u} = \frac{1}{2}\left( m_{u}\dot{{\varvec{CG}}}_{u}^{T}\dot{{\varvec{CG}}}_{u} +{{\varvec{\omega }}}_{u}^{T}I_{u}{{\varvec{\omega }}}_{u}\right) -m_{u}g[0~0~1]{{\varvec{CG}}}_{u}\\ {\mathscr {L}}_{r} = \frac{1}{2}\left( m_{r}\dot{{{\varvec{CG}}}}_{r}^{T}\dot{{{\varvec{CG}}}}_{r} +{{\varvec{\omega }}}_{r}^{T}I_{r}{{\varvec{\omega }}}_{r}\right) ~-m_{r}g[0~0~1]{{\varvec{CG}}}_{r}\\ \end{array} \end{aligned}$$

where $m_{u}$ and $m_{r}$ are the mass, and $I_{u}$ and $I_{r}$ are the inertias of the ulna and the radius in their bone-fixed frames, respectively.

### Integration of the elbow model into the shoulder model

The Lagrangians ${\mathscr {L}}_{u}$ and ${\mathscr {L}}_{r}$ are added to the Lagrangian of the shoulder model (Eq. 9). It includes the Lagrangians of the clavicle (${\mathscr {L}}_c$), the scapula (${\mathscr {L}}_{s}$), and the humerus (${\mathscr {L}}_h$).

9 $$\begin{aligned} {\mathscr {L}} = {\mathscr {L}}_c + {\mathscr {L}}_{s} + {\mathscr {L}}_h + {\mathscr {L}}_{u} + {\mathscr {L}}_{r} \end{aligned}$$

where ${\mathscr {L}}$ is the augmented Lagrangian of the shoulder and elbow model.

Two constraints are also used in the shoulder kinematic model. They force trigonum scapulae (TS) and angulus acromialis (AI) points on the scapula medial boarder to always lie on two ellipsoids. The ellipsoids approximate the ribcage and the underlying soft tissues of each one of TS and AI. The constraints are written as

10 $$\begin{aligned} \begin{array}{ll} \Phi _{\mathrm{TS}}({{\varvec{q}}}(t)) = \left( {}_{t}{{\varvec{TS}}}(t)-{{\varvec{e}}}_{0}\right) ^TE_{\mathrm{TS}}\left( {}_{t}{{\varvec{TS}}}(t)-{{\varvec{e}}}_{0}\right) -1= 0\\ \Phi _{\mathrm{AI}}({{\varvec{q}}}(t)) ~= \left( {}_{t}{{\varvec{AI}}}(t)-{{\varvec{e}}}_{0}\right) ^TE_{\mathrm{AI}}\left( {}_{t}{{\varvec{AI}}}(t)-{{\varvec{e}}}_{0}\right) ~-1= 0 \end{array} \end{aligned}$$

where ${{\varvec{e}}}_{0}$ is the center of the ellipsoids in the thorax frame, and $E_{\mathrm{TS}}$ and $E_{\mathrm{AI}}$ are the matrices corresponding to each of the ellipsoids.

The equations of motion are obtained using the Lagrange’s equations (Eq. 11).

11 $$\begin{aligned} \frac{d}{dt}\left( \frac{\partial {\mathscr {L}}}{\partial \dot{{\varvec{q}}}}\right) - \frac{\partial {\mathscr {L}}}{\partial {{\varvec{q}}}} = \frac{\partial {\Omega }}{\partial \dot{{{\varvec{q}}}}}M + \lambda _{\mathrm{TS}}\frac{\Phi _{\mathrm{TS}}}{\partial {{\varvec{q}}}} + \lambda _{\mathrm{AI}}\frac{\Phi _{\mathrm{AI}}}{\partial {{\varvec{q}}}} \end{aligned}$$

where ${{\varvec{q}}}$ is the generalized coordinate vector of the shoulder and elbow model, and $\Omega$ is a horizontal matrix consisting of the angular velocities of all the 5 bone segments. The vertical matrix *M* includes the muscle resultant moments around each one of the 5 joints. The first term on the right-hand side is the generalized force vector defined as the multiplication of the partial angular velocity matrix ($\frac{\partial {\Omega }}{\partial \dot{{\varvec{q}}}}$) and *M*. The resultant moment matrix *M* can be expressed as $M = W{{\varvec{f}}}$, where *W* is the moment arm matrix and ${{\varvec{f}}}$ is a vector including the magnitudes of all the muscle forces. The Lagrange multipliers $\lambda _{\mathrm{TS}}$ and $\lambda _{\mathrm{AI}}$ are proportional to the magnitudes of the forces applied on the scapula due to the ribcage constraints. The jacobians of the constraints ($\frac{\Phi _{\mathrm{TS}}}{\partial {{\varvec{q}}}}$ and $\frac{\Phi _{\mathrm{AI}}}{\partial {{\varvec{q}}}}$) define the generalized moment arms of the constraints.

**Figure 2:**
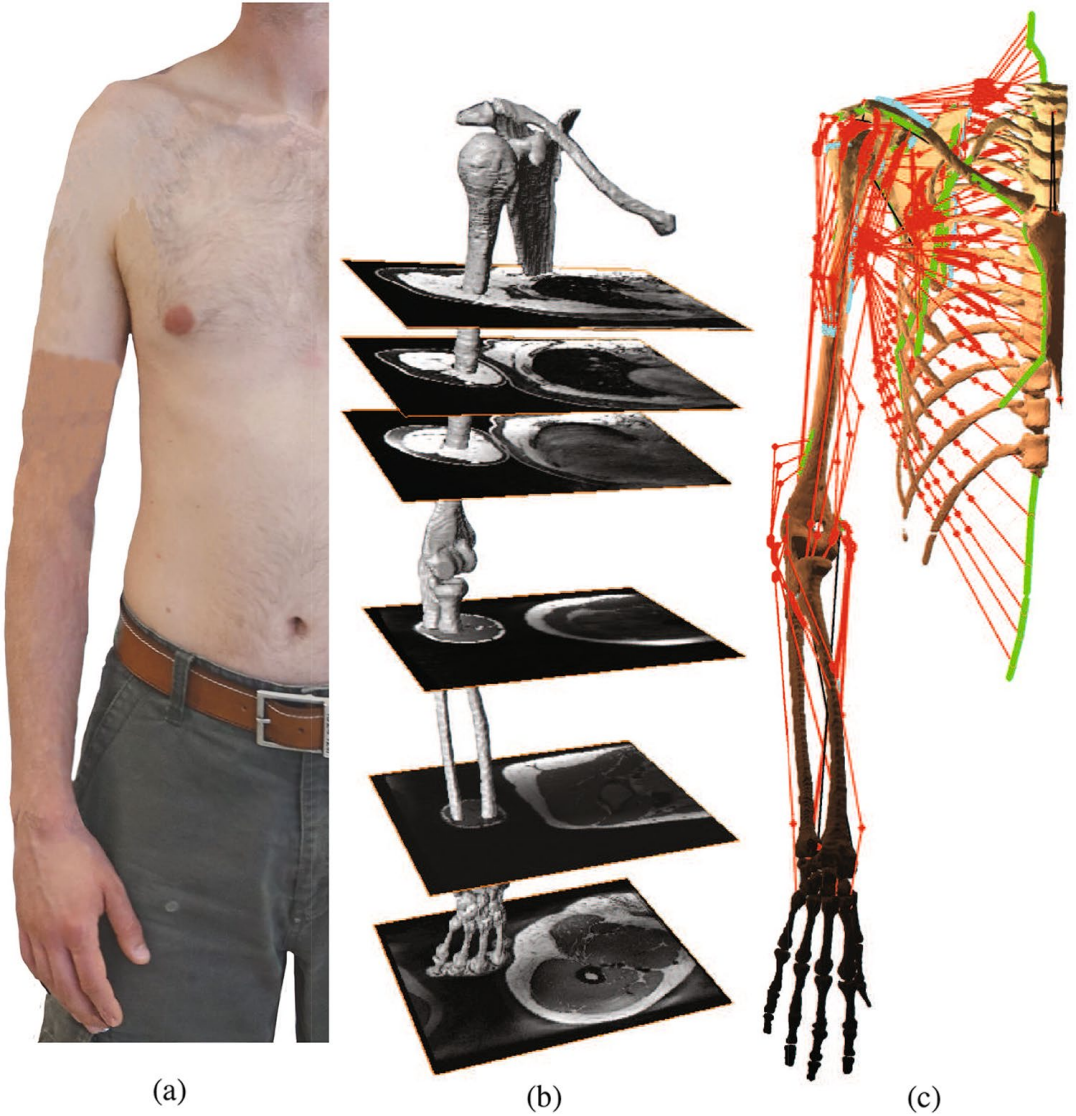
The developed shoulder and elbow model. (**a**) The anthropometric data of a healthy male subject is used to develop the model. (**b**) The bone morphologies and muscles origins/insertions are deduced from MRI scans of the same subject. (**c**) The model consists of thorax, clavicle, scapula, humerus, ulna, radius, and a hand tied to the radius. It has nine DOF represented by eleven generalized coordinates and two holonomic constraints. The model includes 42 muscles that can be represented by up to 20 massless elastic strings. Muscle origins and insertions are denoted by green and blue areas, respectively.

The moment arm matrix *W* can be obtained using its well-known geometric definition or the tendon excursion method^56^. In either ways, the paths taken by the muscles approximated as massless elastic strings are required. To this end, the obstacle set method is used^55^. The origins/insertions and the associated via points of 14 muscles spanning the elbow are defined based on the MRI scans and the help of a professional radiologist. Their associated wrapping objects are set by modifying recommendations of^1^ for the type, center, axis, and radius of the objects to best fit the MRI scans. They include triceps brachii long/medial/lateral, biceps brachii short/long, brachialis, brachioradialis, supinator, pronator Teres, flexor carpi radialis/ulnaris, and extensor carpi radiali long/radialis bervis/ulnaris. Therefore, the shoulder and elbow model consists of 42 muscle groups that each one of them can be represented by up to 20 strings (Fig. 2).

The equations of motion (Eq. 11) are solved for unknown muscle forces ${{\varvec{f}}}$ using inverse dynamics for a given ${{\varvec{q}}}$. The shoulder and elbow model is over-actuated, i.e. there are more muscles (42 times the number of strings used for each muscle) than the number of equations (11 equations, one per generalized coordinate). Therefore, the following so-called standard load-sharing is casted to solve the over-actuation problem using static optimization^67^.

12 $$\begin{aligned} \begin{array}{ll} \min \limits _{\tilde{{\varvec{f}}}} &{}{\tilde{{\varvec{f}}}}^TP{\tilde{{\varvec{f}}}}\\ {\text {s.t.}} &{} \frac{d}{dt}\left( \frac{\partial {\mathscr {L}}}{\partial {\dot{{\varvec{q}}}}}\right) - \frac{\partial {\mathscr {L}}}{\partial {{\varvec{q}}}} = \left[ \frac{\partial {\Omega }}{\partial {\dot{{\varvec{q}}}}}W~ \frac{\Phi _{\mathrm{TS}}}{\partial {{\varvec{q}}}}~ \frac{\Phi _{\mathrm{AI}}}{\partial {{\varvec{q}}}}\right] {\tilde{{\varvec{f}}}}\\ &{}{{\varvec{0}}} \le {\tilde{{\varvec{f}}}} \le {\tilde{{\varvec{f}}}}_{\max }\\ &{}{{\varvec{\psi }}}({{\varvec{q}}},~ {\dot{{\varvec{q}}}}, ~ {\ddot{{\varvec{q}}}}, {\tilde{{\varvec{f}}}}) \le {{\varvec{0}}} \end{array} \end{aligned}$$

where ${\tilde{{\varvec{f}}}} \equiv [{{\varvec{f}}}^T~\lambda _{\mathrm{TS}}~\lambda _{\mathrm{AI}}]^T$, and *P* is a diagonal matrix including the inverse squared of muscles PCSAs. The numerical values for PCSAs are set according to^68^. The constraint ${{\varvec{\psi }}}$ forces the JRF to always point toward inside of a cone that replicates the glenoid fossa. It is commonly called the GH joint stability constraint. The optimization is a quadratic programming problem that can be solved using quadprog of Matlab. It defines ${\tilde{{\varvec{f}}}}$ such that the sum of squared muscle stresses are minimized, while the constraints are satisfied. An EMG-Assisted load-sharing scheme developed in^69^ is also included to the model to account for muscle co-contractions.

### Model scaling

#### Scaling BSIP

The model BSIP are scaled based on the subject’s gender, weight ($m_{B}$), and height ($l_H$) using the 3D predictive equations of^38^. More specifically, the mass and the length of each bone segment is defined as portions of $m_{B}$ and $l_H$, respectively (Table 1). The portions slightly vary for male and female subjects. The resulting bone segment masses and lengths are used to define their inertias in the transverse and the lateral directions of the bone-fixed frames.

**Table 1:** Scaling BSIP based on subject’s gender, weight ($m_{B}$), and height ($l_H$) using the 3D predictive equations of^38^.

	BSIP	Male	Female
Clavicle	$m_c$	$0.18m_{B}$	$0.18m_{B}$
Scapula	$m_{s}$	$0.82m_{B}$	$0.82m_{B}$
Humerus	$m_h$	$2.4m_{B}$	$2.2m_{B}$
	$l_h$	$\frac{27}{177}l_H$	$\frac{24.3}{161}l_H$
	$I_{h_{t}}$	$(0.315l_h)^{2}m_h$	$(0.33l_h)^{2}m_h$
	$I_{h_{l}}$	$(0.14l_h)^{2}m_h$	$(0.17l_h)^{2}m_h$
Ulna	$m_{u}$	$(0.62)(1.7)m_{B}$	$(0.62)(1.3)m_{B}$
	$l_{u}$	$\frac{28.3}{177}l_H$	$\frac{24.7}{161}l_H$
	$I_{u_{t}}$	$(0.275l_{u})^{2}m_{u}$	$(0.255l_{u})^{2}m_{u}$
	$I_{u_{l}}$	$(0.11l_{u})^{2}m_{u}$	$(0.14l_{u})^{2}m_{u}$
Radius	$m_{r}$	$(0.38)(1.7)m_{B}$	$(0.38)(1.3)m_{B}$
	$l_{r}$	$\frac{28.3}{177}l_H$	$\frac{24.7}{161}l_H$
	$I_{r_{t}}$	$(0.275l_{r})^{2}m_{r}$	$(0.255l_{r})^{2}m_{r}$
	$I_{r_{l}}$	$(0.11l_{r})^{2}m_{r}$	$(0.14l_{r})^{2}m_{r}$

Clavicle, scapula, humerus, ulna, and radius masses are denoted by $m_c$, $m_{s}$, $m_h$, $m_{u}$, and $m_{r}$, respectively. The segments lengths of humerus, ulna, and radius are $l_h$, $l_{u}$, and $l_{r}$. Their inertias in transverse and lateral directions of their bone-fixed frames are denoted by $I_{h_{t}}$, $I_{u_{t}}$, $I_{r_{t}}$, $I_{h_{l}}$, $I_{u_{l}}$, and $I_{r_{l}}$, respectively. The $m_{u}$ and $m_{r}$ are defined as 62% and 38% of the forearm weight. The $m_{B}$ and $l_H$ are expressed in centigram and centimeter, respectively.

#### Scaling skeletal morphologies

The skeletal morphologies are scaled using an anisotropic scaling matrix *S*. It is defined according to $l_H$ and the subject’s shoulder width $l_W$ (Eq. 13). The $l_W$ is defined as the distance between the two angulus acromialis landmarks on the left and the right shoulder.

13 $$\begin{aligned} S = \begin{bmatrix} \frac{l_W}{l_{W_g}} &{}\quad 0 &{}\quad 0 \\ 0 &{}\quad \frac{l_W}{l_{W_g}}&{}\quad 0\\ 0 &{}\quad 0&{}\quad \frac{l_H}{l_{H_g}} \end{bmatrix} \end{aligned}$$

where $l_{H_g}$ and $l_{W_g}$ are the height and shoulder width of the generic model.

The two ribcage ellipsoids are scaled by dilating a base ellipsoid. The base ellipsoid is obtained during the construction of the generic model by fitting it to the ribcage. It is centered at ${{\varvec{e}}}_{0}$ with axes equal to $e_{{\text {BE}}_x}$, $e_{{\mathrm{BE}}_{y}}$, and $e_{{\mathrm{BE}}_z}$. The centers of the two dilated ellipsoids are obtained by scaling ${{\varvec{e}}}_{0}$ with *S*. Then, their axes are obtained by an isotropic dilation of the base ellipsoid so that they include their associated scaled landmark TS or AI. The dilation factors ($\delta _{\mathrm{TS}}$ and $\delta _{\mathrm{AI}}$) are calculated by using the scaled TS and AI in the equations of their respective ellipsoids and solving the resulting 6th degree polynomial equations for $\delta _{\mathrm{TS}}$ and $\delta _{\mathrm{AI}}$ (Eq. 14).

14 $$\begin{aligned} \begin{array}{ll} (S{}_{t}{{\varvec{TS}}}-S{{\varvec{e}}}_{0})^T\begin{bmatrix} \frac{1}{(e_{{\mathrm{BE}}_{x}}+\delta _{\mathrm{TS}})^{2}} &{}\quad 0 &{}\quad 0 \\ 0 &{}\quad \frac{1}{(e_{{\mathrm{BE}}_{y}}+\delta _{\mathrm{TS}})^{2}}&{}\quad 0\\ 0 &{}\quad 0&{}\quad \frac{1}{(e_{{\mathrm{BE}}_z}+\delta _{\mathrm{TS}})^{2}} \end{bmatrix}(S_{t}{{\varvec{TS}}}-S{{\varvec{e}}}_{0})-1= 0\\ (S_{t}{{\varvec{AI}}}-S{{\varvec{e}}}_{0})^T\begin{bmatrix} \frac{1}{(e_{{\mathrm{BE}}_{x}}+~\delta _{\mathrm{AI}})^{2}} &{}\quad 0 &{}\quad 0 \\ 0 &{}\quad \frac{1}{(e_{{\mathrm{BE}}_{y}}+~\delta _{\mathrm{AI}})^{2}}&{}\quad 0\\ 0 &{}\quad 0&{}\quad \frac{1}{(e_{{\mathrm{BE}}_z}+\delta _{\mathrm{AI}})^{2}} \end{bmatrix}(S{}_{t}{{\varvec{AI}}}-S{{\varvec{e}}}_{0})-1= 0 \end{array} \end{aligned}$$

**Figure 3:**
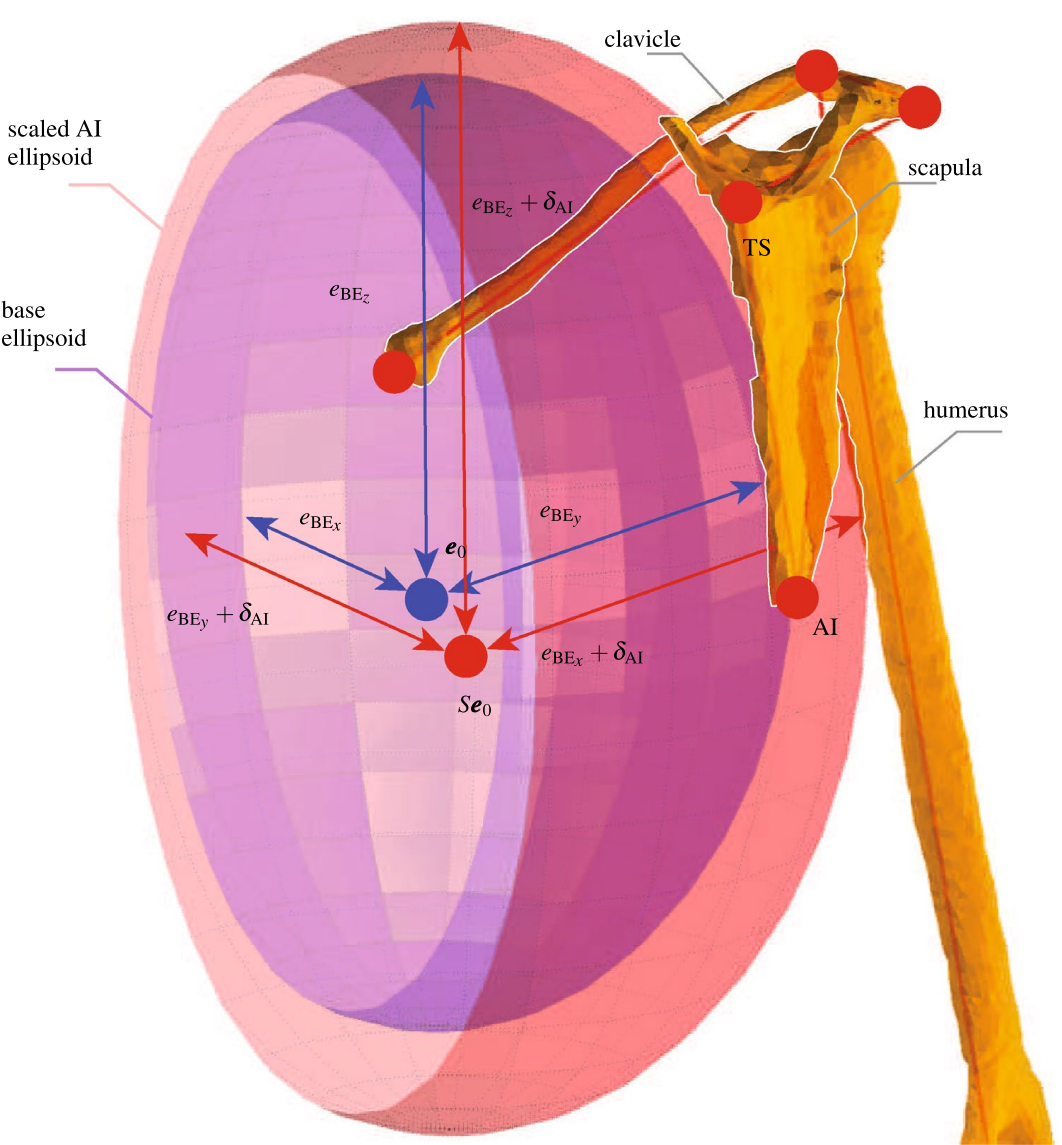
Scaling the ribcage ellipsoid containing AI. A base ellipsoid (blue) is dilated by $\delta _{\mathrm{AI}}$ to obtain the ribcage ellipsoid AI (red). The base ellipsoid is centered at ${{\varvec{e}}}_{0}$ with axes equal to $e_{\mathrm{BE}_{x}}$, $e_{\mathrm{BE}_{y}}$, and $e_{\mathrm{BE}_{z}}$. The center of the scaled AI ellipsoid is also obtained by scaling ${{\varvec{e}}}_{0}$ with *S*. Scaling the TS ribcage ellipsoid follows the same procedure and results in an ellipsoid centered at $S{{\varvec{e}}}_{0}$ and dilated by $\delta _{\mathrm{TS}}$.

Equation 14 is solved numerically for instance using roots of Matlab. It has only two positive roots that are considered as $\delta _{\mathrm{TS}}$ and $\delta _{\mathrm{AI}}$ (Fig. 3).

In a case where videogrammetry measurement data are available for a subject, the scaling matrix *S* is defined for each bone segment. The landmarks on each bone segment are used to define its length. The resulting length is used to define a scaling factor by comparing it to an associated length from the generic model. The scaling of the ribcage ellipsoids follows the same approach as Eq. (14), except that the measured positions of TS and AI are used instead of their scaled positions.

**Figure 4:**
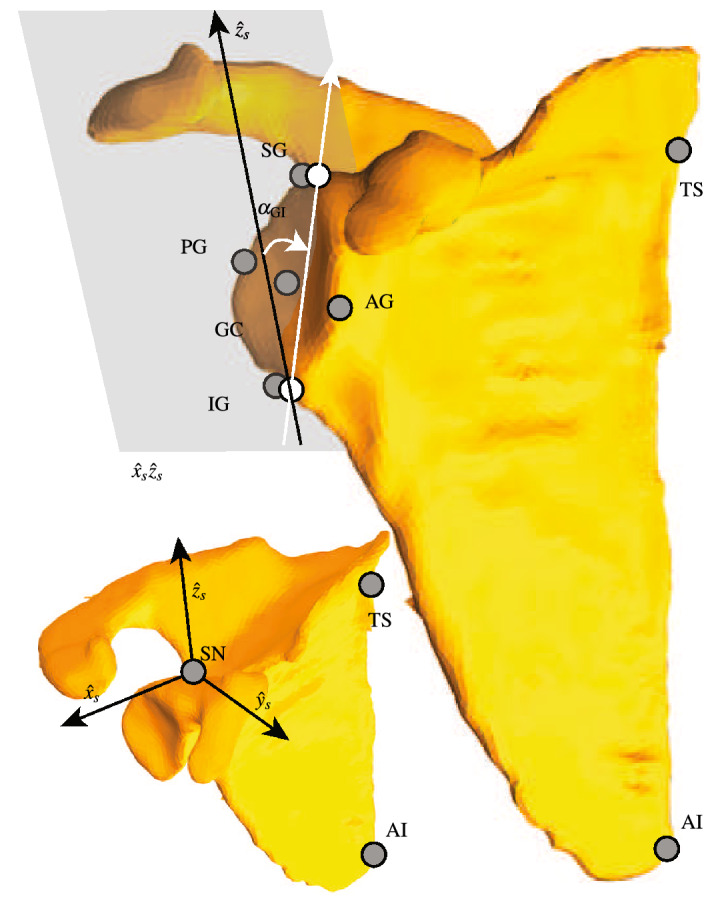
Scaling and definition of the glenoid inclination/version ($\alpha _{\mathrm{GI}}$ and $\alpha _{\mathrm{GV}}$). The scapula frame $\{{\hat{x}}_{s}\;{\hat{y}}_{s}\;{\hat{z}}_{s}\}$ is attached to SN and is defined according to Eq. (15). The $\alpha _{\mathrm{GI}}$ is defined in ${\hat{x}}_{s}{\hat{z}}_{s}$ plane where the two points IG and SG are projected (white circles). It is defined as the angle between the ${\hat{z}}_{s}$ and a vector passes through the two projected points of IG and SG. The $\alpha _{\mathrm{GV}}$ has a similar definition, but in the ${\hat{x}}_{s}{\hat{y}}_{s}$ plane and through projections of PG and AG. The adaptation of $\alpha _{\mathrm{GI}}$ and $\alpha _{\mathrm{GV}}$ results in a scaled GC point that modifies the cone of the stability constraint (Eq. 12).

The glenoid inclination($\alpha _{\mathrm{GI}}$) and version ($\alpha _{\mathrm{GV}}$) are determined according to a definition provided in^44,45^. A bone-fixed frame attached to the spino-glenoid notch (SN) defines scapula configurations (Eq. (15) and Fig. 4).

- Scapula bone-fixed frame: $\{{{\varvec{O}}}_{s},{\hat{x}}_{s},{\hat{y}}_{s},{\hat{z}}_{s}\}$
  15 $$\begin{aligned} \begin{array}{ll} {{\varvec{O}}}_{s} \equiv {}_{t}{{\varvec{SN}}}\\ {\hat{x}}_{u} = {\hat{y}}_{s}\times {\hat{z}}_{s}\\ {\hat{y}}_{s} = ({}_{t}{{\varvec{SN}}} - {}_{t}{{\varvec{AI}}})\times ({}_{t}{{\varvec{SN}}} - {}_{t}{{\varvec{TS}}}) \implies {\hat{y}}_{s} = {{\hat{y}}_{s}}/{|{\hat{y}}_{s}|}\\ {\hat{z}}_{s} = ({}_{t}{{\varvec{SN}}} - {}_{t}{{\varvec{TS}}})\times {\hat{y}}_{s}, \implies {\hat{z}}_{s} = {{\hat{z}}_{s}}/{|{\hat{z}}_{s}|}\\ {}^t_{s}R = [{\hat{x}}_{s}~{\hat{y}}_{s}~{\hat{z}}_{s}] \end{array} \end{aligned}$$

The $\alpha _{\mathrm{GI}}$ is the angle between the ${\hat{z}}_{s}$ and a vector connecting the inferior glenoid (IG) and the superior glenoid (SG) points projected on ${\hat{x}}_{s}{\hat{z}}_{s}$ plane. The $\alpha _{\mathrm{GV}}$ is the angle between the ${\hat{y}}_{s}$ and a vector connecting the posterior glenoid (PG) and the anterior glenoid (AG) points projected on ${\hat{x}}_{s}{\hat{y}}_{s}$ plane. Provided subject specific values for $\alpha _{\mathrm{GI}}$ and $\alpha _{\mathrm{GV}}$, their associated angles can be adapted in the generic model. To this end, first $\Delta \alpha _{\mathrm{GI}}=\alpha _{\mathrm{GI}}-\alpha _{{\mathrm{GI}}_g}$ and $\Delta \alpha _{\mathrm{GV}}=\alpha _{\mathrm{GV}}-\alpha _{{\mathrm{GV}}_g}$ are defined, where subindex *g* denotes the values of the generic model. The rotation operators $R_{\mathrm{GI}}$ and $R_{\mathrm{GV}}$ are used according to Eq. (16) to rotate the glenoid center (GC) around ${\hat{y}}_{s}$ and ${\hat{z}}_{s}$ by $\Delta \alpha _{\mathrm{GI}}$ and $\Delta \alpha _{\mathrm{GV}}$, respectively. The resulting GC is used to construct a cone frame that defines the GH joint stability constraint (Eq. 12).

16 $$\begin{aligned} \begin{array}{ll} R_{\mathrm{GI}} ~= {\hat{y}}_{s}{\hat{y}}_{s}^T+\cos {\Delta \alpha _{\mathrm{GI}}}\left( I- {\hat{y}}_{s}{\hat{y}}_{s}^T\right) ~+\sin {\Delta \alpha _{\mathrm{GI}}} [{\hat{y}}_{s}]\\ R_{\mathrm{GV}} = {\hat{z}}_{s}{\hat{z}}_{s}^T+\cos {\Delta \alpha _{\mathrm{GV}}}\left( I- {\hat{z}}_{s}{\hat{z}}_{s}^T\right) +\sin {\Delta \alpha _{\mathrm{GV}}}[{\hat{z}}_{s}] \end{array} \end{aligned}$$

where the cross product matrices corresponding to ${\hat{y}}_{s}$ and ${\hat{z}}_{s}$ are denoted by $[{\hat{y}}_{s}]$ and $[{\hat{z}}_{s}]$, respectively.

#### Scaling muscles architectures

The muscles insertion/origins, via points, and wrapping objects’ radii and centers are all scaled using the matrix *S*. In a case where videogrammetry measurement data are available for a subject, the muscle architectures are scaled using the scaling matrix *S* of their corresponding bone segment.

The muscle PCSAs are scaled based on the subject’s gender and body muscle-percentage ($r_m$). Predictive equations^61^ are used that provide an estimation of $r_m$ based on subject’s BMI defined as ${\text {BMI}}\equiv \frac{m}{l^{2}}$ (Eq. 17). The ratio of the subject’s $r_m$ to that of the generic model is used to scale PCSAs. Furthermore, the model allows neglecting the scaled PCSAs in case subject specific values are provided.

17 $$\begin{aligned} r_m = \left\{ \begin{array}{ll} 1.09 - 0.0149{\text {BMI}} + 0.00009{\text {BMI}}^{2} &{} {\text {male}}\\ 1.08 - 0.0203{\text {BMI}} + 0.000156{\text {BMI}}^{2} &{} {\text {female}} \end{array} \right. \end{aligned}$$

### Shoulder and elbow Matlab toolbox

The developed shoulder and elbow model provides predictions of muscles and joints reaction forces for a measured motion. It is developed on open-source principles in Matlab. It has a graphical user interface (GUI) that facilitates its applications. It has 5 main sub-tool windows that are briefly described below (Fig. A1–A7 of the appendix available as online supplementary material).

1. Subject specific tool
  It allows scaling or adapting the BSIP, skeletal morphologies, and muscles architectures of the generic model using subject’s gender, height, shoulder width, and weight. The scaled model can be compared visually to the generic model (Fig. A2).
2. Muscle wrapping tool
  It allows visual verifications of the muscles paths during different joint configurations. The muscles insertions/origins, wrapping objects’ centers and radii, and via points can be verified (Fig. A3).
3. Kinematics tool
  It allows reconstructing a motion of the upper extremity with or without videogrammetry measurement data (Fig. A4). Its first sub-tool window calculate joints angles evolutions in the lack of measurement data for a desired initial and final configuration of the upper extremity^63^. The second sub-tool can construct a measured motion using multi-segment optimization method and provide joints angles evolutions^70^.
4. Moment arms tool
  For the joint angles evolutions obtained from the kinematics tool, it provides muscles moment arms (Fig. A5). The muscles moment arms are calculated using both the geometrical and the tendon excursion methods and can be compared.
5. Force prediction tool
  It provides predictions of muscles forces and JRF using inverse-dynamics and optimal load-sharing (Fig. A6). It also allows inclusion of electromyography (EMG) data and force predictions with or without the GH joint stability constraint.

### Parameter study

Subject’s gender, height, weight, glenoid inclination, and PCSAs of RC muscles are independently varied to evaluate their effects on the JRF predictions. Two variations are considered for the height, 1.60 m and 1.95 m. The weight variations are 60 kg and 100 kg. The glenoid inclination is varied from its generic value of $7^{\circ }$ to $-7^{\circ }$ and $15^{\circ }$, according to the variations observed in healthy subjects^71^. The PCSAs of RC muscles are reduced by 50%^72^. The resulting PCSAs are 10.42 ${\hbox{cm}}^{2}$, 16.66 ${\hbox{cm}}^{2}$, 17.84 ${\hbox{cm}}^{2}$, and 3.40 ${\hbox{cm}}^{2}$ for supraspinatus (Ss), infraspinatus (Is), subscapularis (Sc), and teres minor (Tm), respectively. The generic model is scaled for each one of the variations. An abduction motion in the scapula plane is simulated using the kinematics tool of the developed toolbox. The results are presented in terms of the JRF predictions along the arm abduction angle.

### Ethics approval and consent to participate

This study was approved by the Center for Biomedical Imaging (CIBM, Protocol number 20130328b), and complied with the principles laid down in the Declaration of Helsinki for experiments involving humans. Institutional ethics committee approval was not required for a pilot study using MRI sequences applied in routine clinical practice. Written informed consent was obtained from the volunteer.

## Results

**Figure 5:**
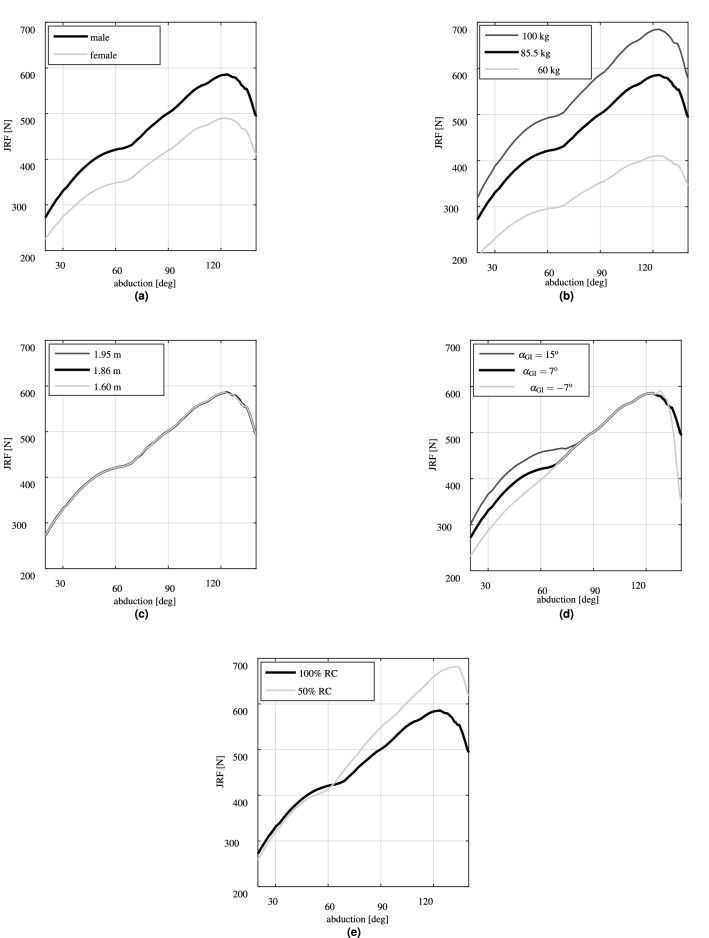
Evaluations of the effects of subject specific parameters on the JRF predictions during abduction motion in the scapula plane. (**a**) Gender, (**b**) weight, (**c**) height, (**d**) glenoid inclination, and (**e**) 50% reduction in PCSAs of RC muscles.

The change of subject’s gender to female resulted in an almost − 95 N shift in the JRF with respect to the male subject of the generic model (Fig. 5a). The JRF predictions for the male generic model increased from 276 N at 20$^{\circ }$ abduction to 585 N (69.80% body weight) at 122$^{\circ }$ abduction and decreased afterward.

The reduction of subject’s weight to 60 kg considerably reduced (almost 30%) the maximum predicted JRF compared to the generic model (Fig. 5b). The JRF increased to 684 N (almost 17%) due to increase in the subject’s weight to 100 kg.

The variations of subject’s height had almost zero effect on the JRF predictions (Fig. 5c).

The variations of the $\alpha _{\mathrm{GI}}$ had negligible effects on the peak of JRF predictions (Fig. 5d). However, its reduction to $-\,7^{\circ }$ resulted in almost 10% less JRF until $60^{\circ }$ abduction. The JRF reduction continued again after $125^{\circ }$ abduction for $\alpha _{\mathrm{GI}}= -7^{\circ }$. For $\alpha _{\mathrm{GI}}= 15^{\circ }$, the JRF at 60$^{\circ }$ abduction increased from 422 N of the generic model to 456 N (almost 8%).

The 50% reduction of PCSAs of RC muscles had negligible effects (less than 3%) on the JRF until 60$^{\circ }$ abduction (Fig. 5e). But, it caused the JRF to increase afterward. The maximum JRF increased to 682 N (less than 17%) and occurred at slightly higher abduction angles (130$^{\circ }$) compared to the generic model.

## Discussion and conclusions

The aim of this study was to develop a scaled-generic musculoskeletal model of shoulder and elbow. The elbow was incorporated into our previously developed shoulder model using two non-perpendicular hinge joints. Fourteen muscles spanning the elbow and shoulder were included in the model. Scaling routines were developed to scale the model’s BSIP, skeletal morphologies, and muscles architectures to a specific subject. The model was developed on open-source principles as a Matlab toolbox. We specifically evaluated the effects of subject’s gender, weight, height, glenoid inclination, and reductions in PCSAs of RC muscles on the JRF predictions during an abduction motion. The JRF prediction of the generic model was consistent with the in vivo measurements of the instrumented prosthesis^73^ and other numerical studies^2,7,9^.

The predicted JRF at 60$^{\circ }$, 90$^{\circ }$, and 120$^{\circ }$ were at most only 13% less than those of the instrumented prosthesis.

The reduction in JRF due to changing the subject’s gender to female was expected. Because, it scaled down the arm weight (from almost 3.51 kg to 2.99 kg by around 15%). It also resulted in a slightly lower $r_m$ (from 77.67 to 67.36%) that reduced the PCSAs.

The weight had the most significant effect among the other parameters. Each extra Kilogram of subject’s weight scaled up the arm weight by almost 1.17%. Therefore, the increase/decrease of the JRF due to increase/decrease of subject’s weight was predictable. This effect would be faded away however, if the JRF was presented in body weight percentage. For instance, both variations of subject’s weight together with the generic model resulted in JRF almost equal to 70% of body weight at 90$^{\circ }$ abduction.

The height had almost no effect on the JRF. The increase of the subject’s height linearly increased the arm weight moment arm. The muscle moment arms were also equivalently increased due to scaling up the wrapping objects. Therefore, these two effects would cancel out each other, given that the changes in the inertial properties and the joint kinematics due to height were negligible.

The more downward the glenoid inclination was, the lower JRF was predicted until 60$^{\circ }$ abduction. This was consistent with the previous studies regarding the glenoid inclination^43–45^. Given that the GH joint was less stable for the beginning of abduction^74^, the downward inclinations of the glenoid fossa could stabilize the joint by centering the JRF within the stability cone. Therefore, lower JRF was predicted. After 60$^{\circ }$ the joint was inherently more stable, provided by the scapula upward rotation. Therefore, the downward inclination of the fossa had negligible effects. The joint became less stable at the end of the abduction^15,75^, where the downward inclination could again play its stabilizing role.

We intuitively expected that the 50% reduction of the RC muscles PCSAs would increase the JRF until 60$^{\circ }$ abduction. Because, the impaired RC muscles supposedly could not perform their stabilizing task, and instead other muscle groups would carry out the task, but with less efficiency. However, the results predicted a slight decrease in JRF until 60$^{\circ }$ abduction. This could be explained by the fact that 50% reduction would not avoid RC muscles from performing their stabilizing task. It has been shown by several studies that the contributions of RC muscles during an abduction motion were limited to less than 50 N^2,67,76^. Therefore, according to the Fick law^77^, they roughly required less than 2 ${\hbox{cm}}^{2}$ PCSAs to carry out their stabilizing task.

A parameter study typically required participants with different anthropometric parameters^31^. For instance, to evaluate the effect of height, several participants with different heights but ideally same weights were required^18^. To predict effects of kinematics on JRF predictions, kinematics of several subjects during a motion were required to be recorded^35,78^. However, a strength of the developed toolbox was that it allowed performing the parameter study, while no participants was required. Because, the toolbox could numerically produce the kinematics associated to each virtual subject considered^63^. Provided by the model GUI, it also exempted the user from cumbersome programming.

One limitation of this study was that a fix carrying angle was considered for the elbow. The elbow carrying angle is the angle between the forearm and the humerus longitudinal axes. It was shown to vary during forearm motion^14^. A fixed carrying angle would therefore compromise the elbow kinematics during its task oriented motions^14,79^. However, given that our model mainly focused on shoulder studies, this simplification could be justified. The other limitation was that the model underestimated the JRF compared to the instrumented prosthesis. This could be expected given that muscles co-contractions were not accounted for in the performed parameter study. It is well-known that the standard load-sharing approach overlooked the muscle co-contractions^76,80^. Future parameter study would leverage the capability of the model to incorporate electromyography (EMG) data to enforce muscle co-contractions^69^. The third limitation referred to the fact that the model only included ideal joints with no translations. However, the GH joint translations play a crucial role in the joint functions and its stability mechanism^81^. Therefore, future developments would allow the model to predict GH joint translations by taking advantage of a framework that has been developed in our group^46^.

In the commercial musculoskeletal packages, when videogrammetry data are available, the model scaling is performed through an optimization procedure. In this optimization, the bone segments are scaled such that the generic model landmarks match the measured markers. The user of these commercial packages must decide which model landmarks should be considered fixed and what landmarks have to be scaled through the optimization. Compared to our scaling approach, this allows the expert users improve model scaling and correct the uncertainties in marker placements. However, this approach may carry the risk of transforming model scaling into model tuning, removing true model uncertainties instead of measurement uncertainties^41^.

Furthermore, we investigated the effects of each parameter separately. More thorough sensitivity analyses could be used to also show compound effects of parameters. A compromise of three factors namely sensitivity, variability, and measurability could then be used to decide whether a parameter must be personalized in a model. For instance a highly sensitive parameter with very little variability which is also difficult to measure could be excluded from subject specific parameters. Finally, this study mainly dealt with presenting the toolbox and the underlying methodologies of its development. Indeed, further investigations were required to evaluate its predictions. It would also be necessary to compare its predictions with other existing free^82^ or commercial^83^ shoulder musculoskeletal packages.

In conclusion, we enhanced the realism and facilitated applications of an existing shoulder model by three main improvements. First, the elbow and the muscle groups spanning the elbow were included in the model. Second, scaled-generic attributes were added to the resulting shoulder and elbow model. The model was finally developed as a Matlab toolbox with a GUI that greatly facilitated its applications. We showed the effects of subject specific parameters on the JRF predictions. Given their considerable effects, it was concluded that their adaptation to each subject could enhance the realism of the model predictions. This work was a step toward subject-specific modeling of shoulder and elbow. In a next step the toolbox would be populated with data from pre and post operative patients for clinical applications related to treatments of the GH joint osteoarthritis with total shoulder arthroplasty.

## Supplementary information

Supplementary Information.

## Data Availability

The datasets used and/or analyzed during the current study are available through https://c4science.ch/source/msm_ul/.
