# Supplementary material for: A Matlab toolbox for scaled-generic modeling of shoulder and elbow
Source: Sci Rep. 2021 Oct 21;11:20806. doi: 10.1038/s41598-021-99856-y (PMC8531442; doi:10.1038/s41598-021-99856-y)
Supplement: Supplementary file 1 — Supplementary Information. [file 41598_2021_99856_MOESM1_ESM.pdf]

A Appendix

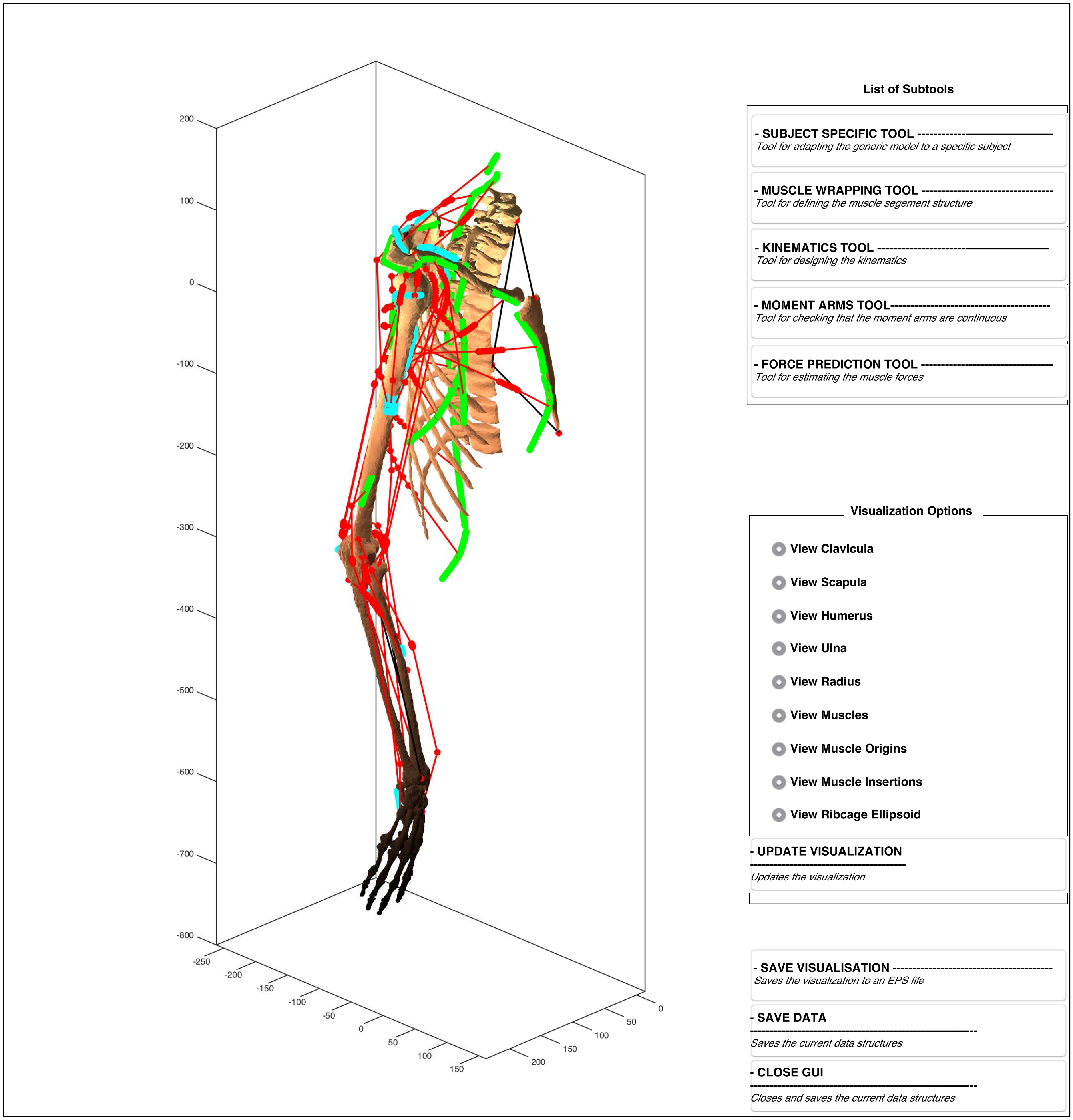

Figure A.1. Illustration of the main window of the developed shoulder and elbow Matlab toolbox. This window provides access to the 5 main sub-tools. It also allows customizing the visualization. Each window is equipped with a save and close push bottoms.

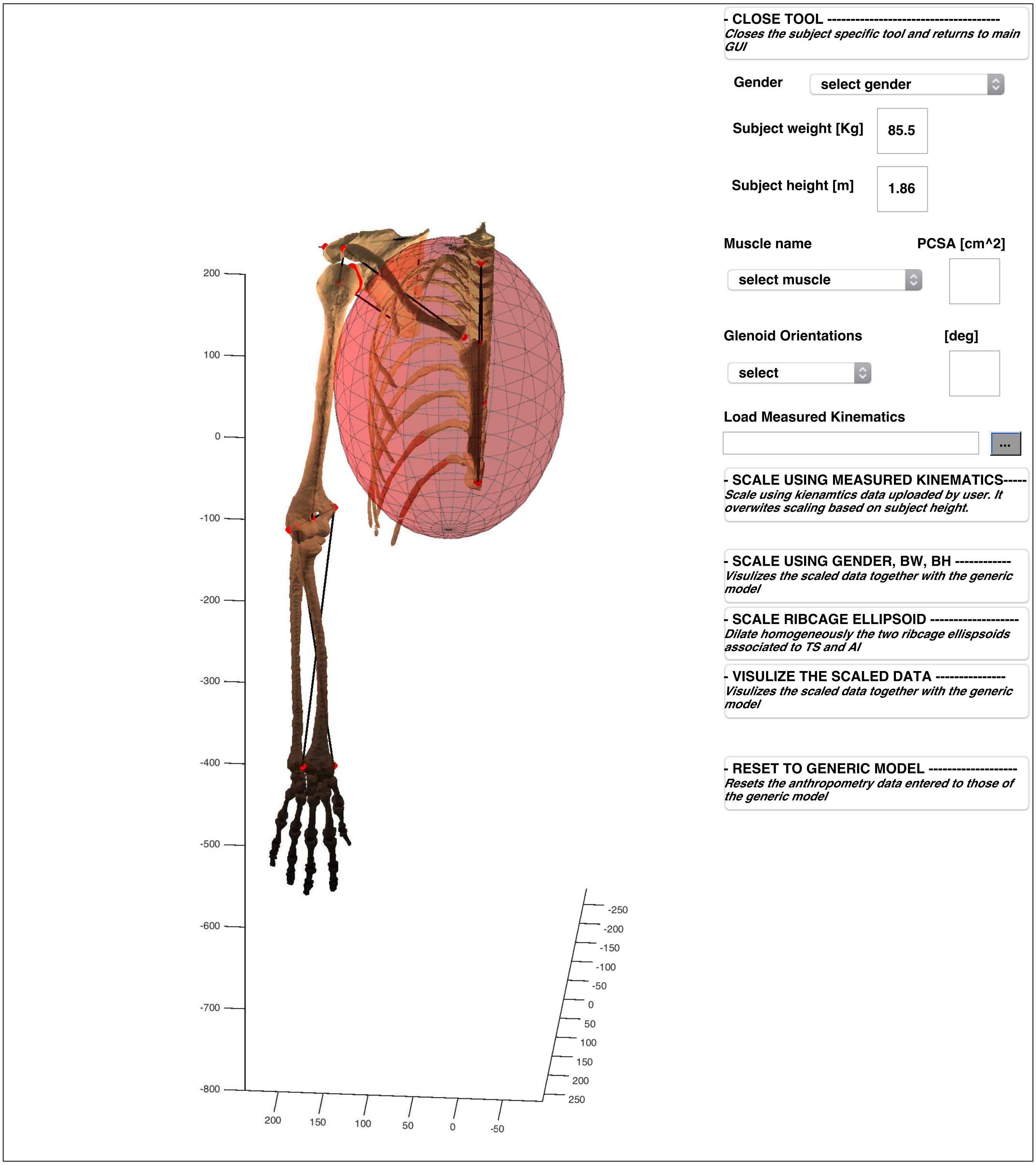

Figure A.2. The subject specific toolbox allows the user to scale the generic model through adapting subject's gender, height, weight, muscles PCSAs, and glenoid orientations (inclination and version). It also allows importing and scaling the model using kinematic measurement data in terms of trajectories of palpated landmarks. The model's BSIP, skeletal morphologies, and muscles architectures are scaled here. The scaled model can be visually compared to the generic model. The option for discarding the changes is indeed provided to set back the generic model.

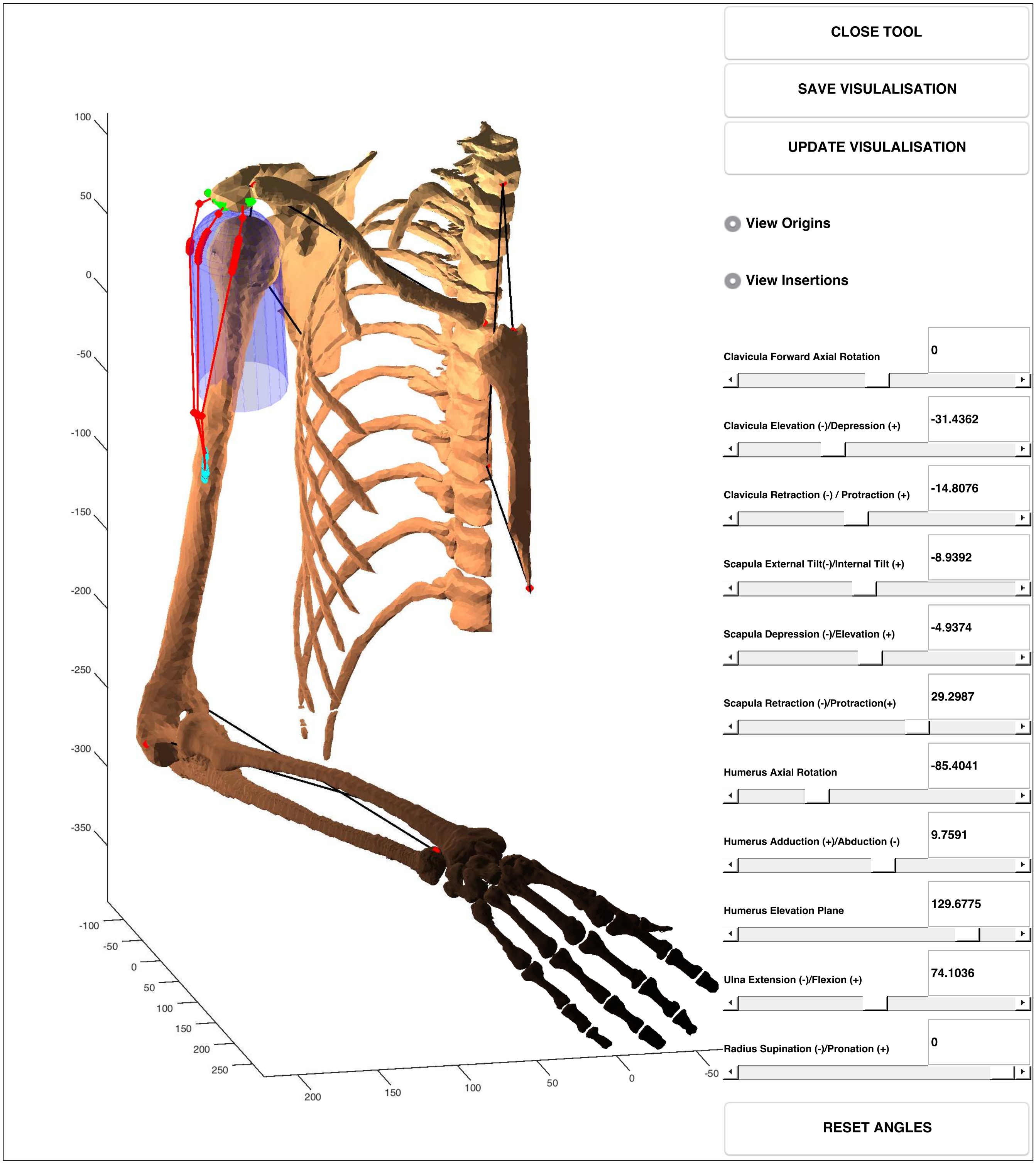

Figure A.3. The muscle wrapping tool provides an interactive environment for visual verification of the muscle paths. The wrapping obstacles, via points, the origin (green), and the insertion (blue) can be checked. Different joint angles can be also imposed to perform the verification in different configurations.

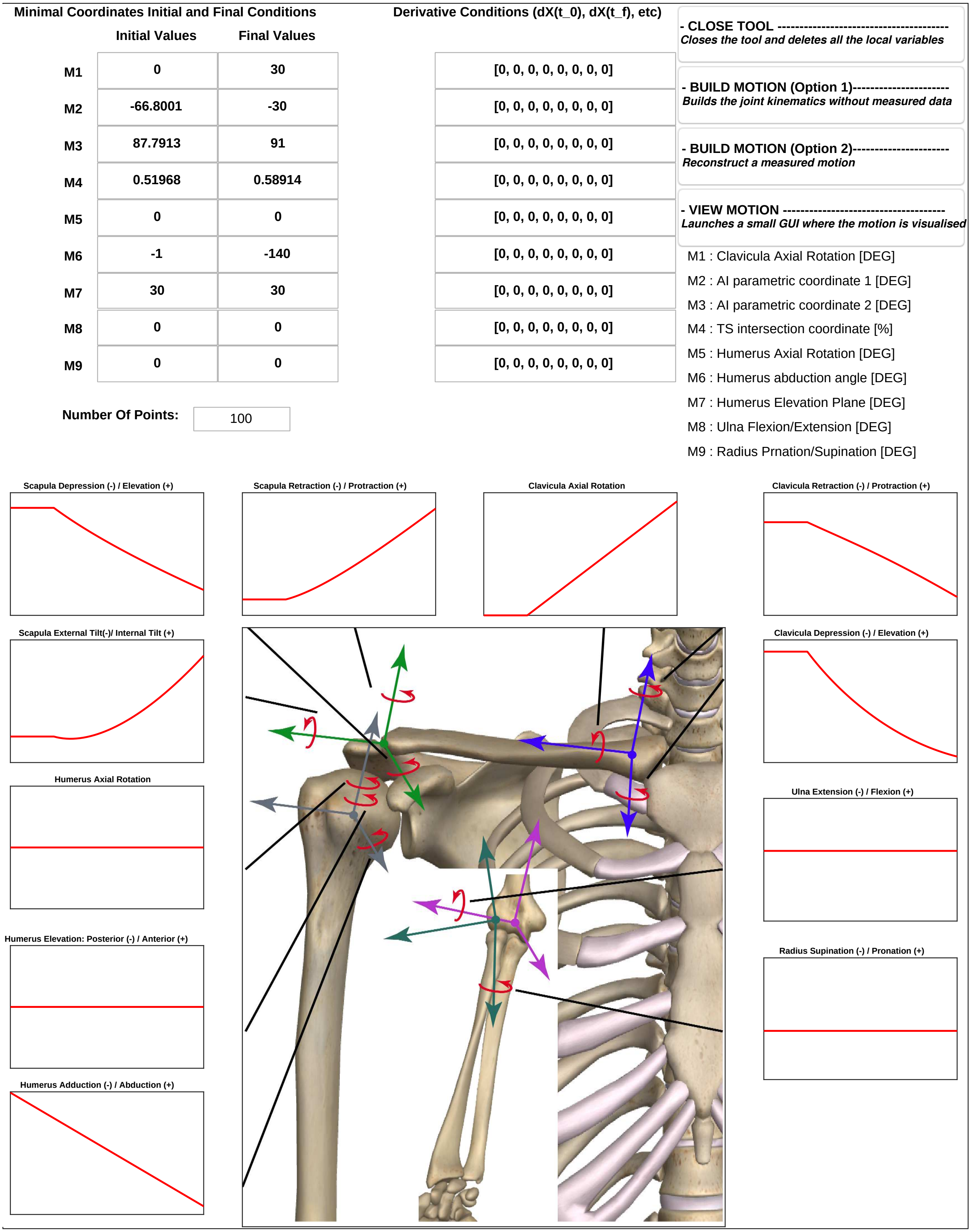

Figure A.4. The kinematics tool allows producing a motion for the shoulder and elbow through two different options. The first option does not require measurement data and is useful for conceptual studies such as sensitivity analysis. The second option allows reconstruction of a measured motion using videogrammetry systems in terms of trajectories of palpated landmarks (Fig. A.5).

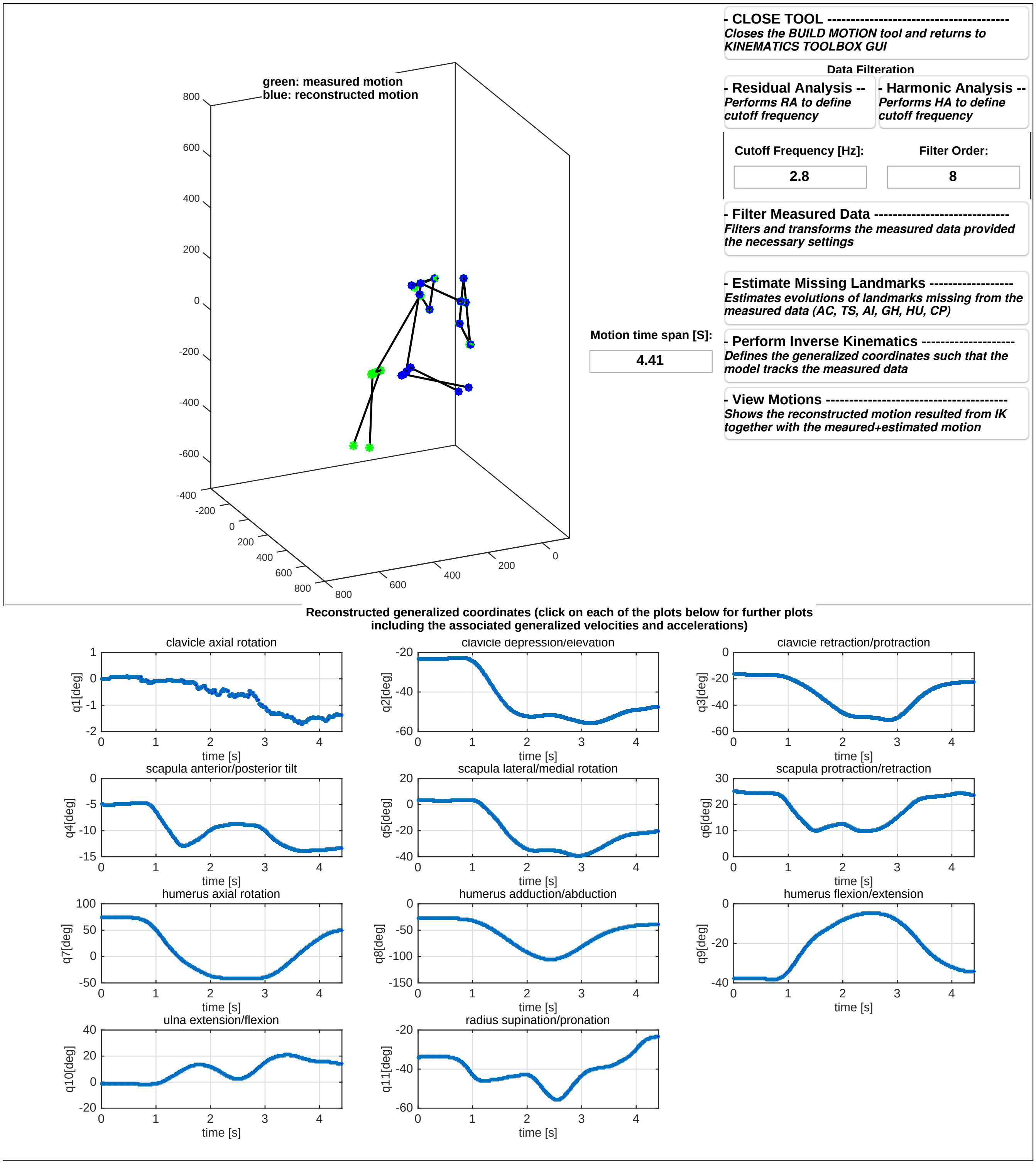

Figure A.5. The second option of the kinematics tool uses the imported measurements from the subject specific tool. It analyzes and filters the measured data. It provides estimations of the missing landmarks and scapula kinematics, even if it is not measured explicitly using scapula kinematic measurement devices. It reconstructs the motion in terms of the joint angles using multi-segment optimization (inverse kinematics). The constructed motion can be animated and saved.

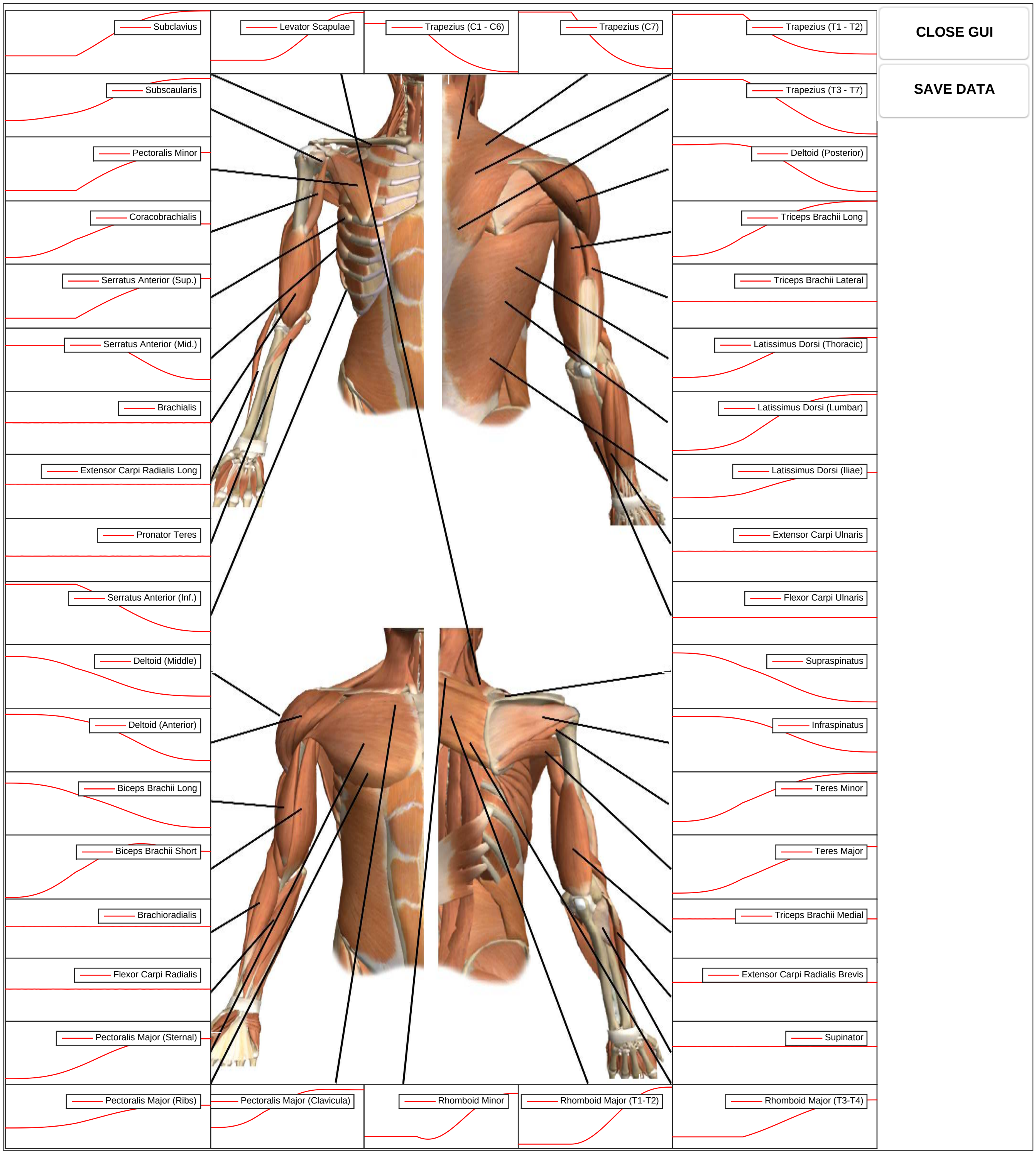

Figure A.6. The moment arms tool calculates the moment arms of the muscles for the simulated motion. The interactive design of this tool allows the user to obtain further details regarding the moment arms of each muscle by a single click. The moment arms around different joints and the muscle length are included for each muscle.

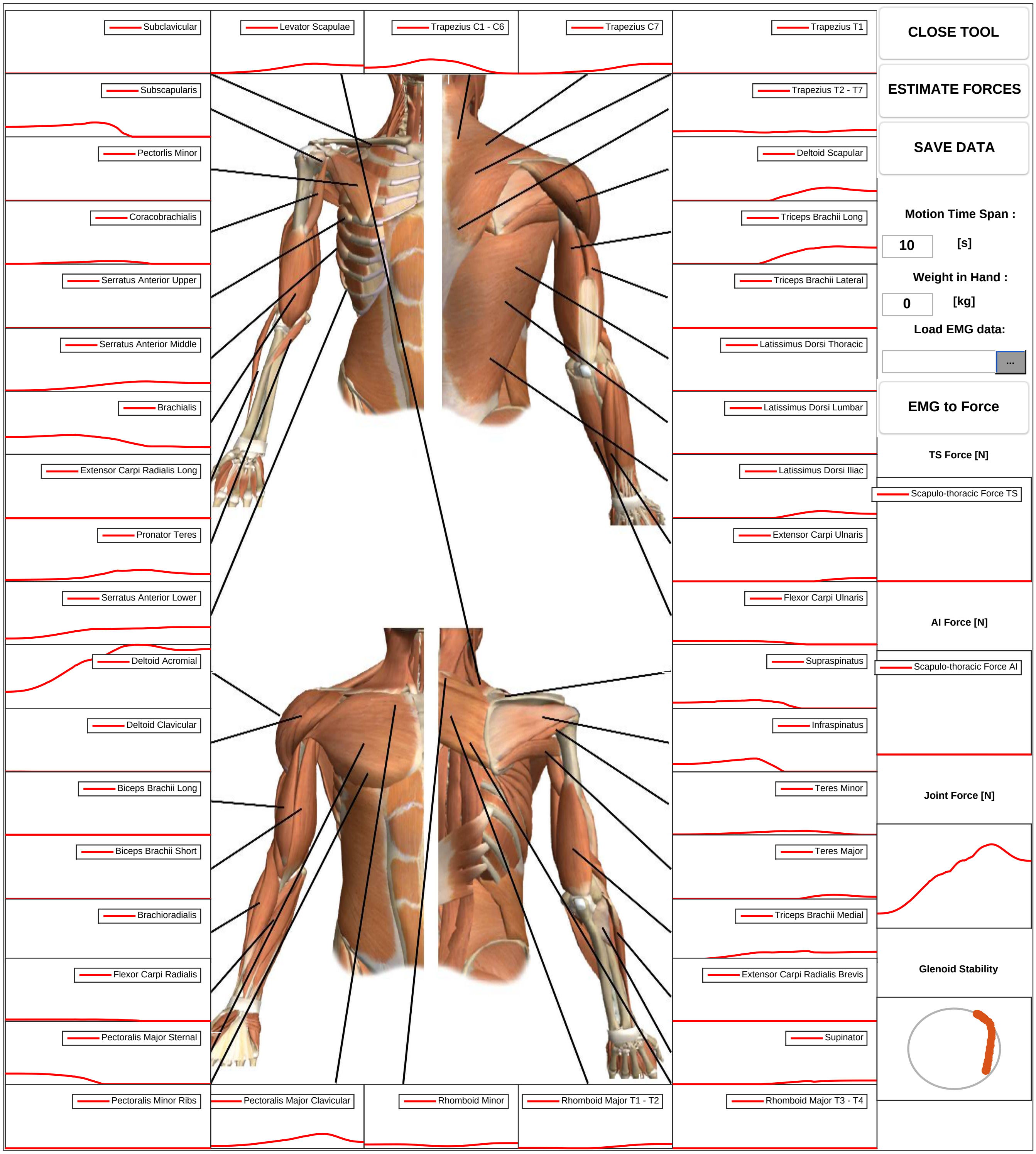

Figure A.7. Finally, the force prediction tool provides predictions of the muscle forces and JRF for the simulated motion. The subject can alternate the speed of the motion and also the weight that the subject carries during the motion. It also includes EMG-assisted load-sharing approach of the model that is not discussed in this study.
